# Study on Mechanical Properties and Microstructure of 2024 Aluminum Alloy Cross-Welded Joint by Friction Stir Welding

**DOI:** 10.3390/ma18102223

**Published:** 2025-05-12

**Authors:** Yanning Guo, Wenbo Sun

**Affiliations:** 1 School of Civil Engineering, Xi’an University of Architecture and Technology, Xi’an 710055, China; 2 School of Aeronautics, Northwestern Polytechnical University, Xi’an 710072, China; wenbo_sun@mail.nwpu.edu.cn

**Keywords:** friction stir welding, cross-welded joint, mechanical properties, residual stress, microstructure

## Abstract

The integral welded panel represents a highly promising aircraft structural component, owing to its lightweight design and reduced connector requirements. However, the complexity of its welded structure results in the formation of cross-welded joints. This study systematically investigated the mechanical properties of the cross-welded joints through tensile tests across different welded regions, which were complemented by fracture morphology examination via scanning electron microscopy (SEM). The residual stress distribution was characterized using X-ray diffraction, while electron backscatter diffraction (EBSD) analysis was used to elucidate the relationship between residual stress and microstructure. Key findings revealed that the cross-welded zone exhibited lower yield strength and ductility than the single-welded zone, and the advancing heat-affected zone demonstrated superior tensile properties relative to the retreating side. Residual stress analysis showed that the cross-welded joint lacked the “double peak” profile characteristic and displayed lower maximum residual stress than the single-welded joint. EBSD analysis indicated significant grain elongation in the cross-welded zone due to mechanical forces during the welding process, resulting in higher dislocation density and deformation, corresponding with elevated residual stress levels.

## 1. Introduction

**Figure 1:**
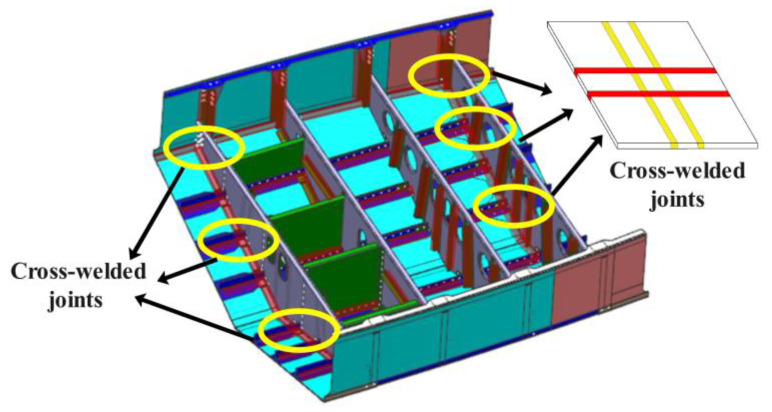
The integral welded panel.

The aviation industry’s growing demands for lightweight structures and enhanced operational reliability have driven significant advancements in aircraft manufacturing technologies [1]. Integral welded panels have emerged as a competitive alternative to mechanically fastened panels, demonstrating notable cost efficiency and weight-saving benefits [2]. However, their structural complexity inevitably introduces cross-welded joints during manufacturing (Figure 1). While existing research has established fundamental performance for conventional welded joints, the unique stress states and microstructural interactions in the cross-welded structures necessitate a comprehensive investigation [3].

As a solid-state joining technology, friction stir welding (FSW) is recognized for its advantages of producing welds with excellent mechanical properties and minimal deformation, making it an important technique in the manufacture of integral welded panels [4]. This process induces complex thermomechanical interactions, where localized temperature gradients and heterogeneous plastic deformation generate significant residual stresses through strain incompatibilities in the weld [5,6]. Residual stress fields not only compromise structural stability but also act as critical determinants of fatigue crack initiation and fracture toughness [7]. Murphy et al. [8,9] elucidated the role of residual stress, demonstrating that while tensile components provide limited load redistribution benefits, the inherent self-equilibration inevitably creates detrimental compressive regimes that degrade structural yield strength. Therefore, it is necessary for precise residual stress management, particularly in multi-pass cross-welded structures where stress superposition effects amplify mechanical property anisotropy [10].

The X-ray method is a mature and widely used stress measurement technique, offering high precision without altering the material state [11,12]. In conventional FSW single-weld, residual stress distributions exhibit a “double peak” profile along the weld direction, with maximum tensile stresses localized at the weld boundary regions [13]. This asymmetric stress patterning arises from differential thermomechanical responses between the welded zone, where the advancing side experiences enhanced thermal input due to synergistic alignment between tool rotation and welding direction [14]. Lee [15] investigated the residual stress distributions of a T-weld and revealed that transverse residual stresses near butt weld intersections decrease by 0–50 MPa due to compressive stress fields from cross welds.

Furthermore, many scholars emphasize the critical interplay between residual stress states and microstructural evolution in determining the weldment performance [16,17]. Microstructural analyses reveal four distinct zones in FSW joints: the dynamically recrystallized welded zone (WZ), the plastically deformed thermo-mechanically affected zone (TMAZ), the thermally modified heat-affected zone (HAZ), and the unaffected base metal (BM) [18]. Advanced characterization through electron backscatter diffraction (EBSD) exposes fundamental structural divergences: the TMAZ demonstrates severe grain elongation with abundant low-angle grain boundaries, indicative of plastic strain accommodation through dislocation glide [19,20]. Conversely, the WZ features fine equiaxed grains with high-angle boundaries, a signature of complete dynamic recrystallization during intense thermomechanical processing [21].

The thermomechanical cycling inherent to cross-welding processes profoundly modifies mechanical properties through repeated heat input [22,23]. Ghiasvand’s systematic investigation revealed that multi-pass welding configurations achieve 18–22% greater tensile-shear force uniformity compared to single-pass welds, demonstrating enhanced load redistribution capabilities [24]. Xu [25] indicated that the ultimate tensile strength and total elongation of the WZ in multi-pass welding were 5% and 17%, respectively, higher than in single-pass welding, indicating simultaneous improvements in both strength and plasticity. Previous analyses of the multi-pass welded joints suggest that the enhancements in mechanical performance can be attributed to the weld grains undergoing repeated deformation and recrystallization, resulting in a more uniform structure [26,27]. However, fundamental understanding remains incomplete regarding the unique microstructure of the cross-welded joints and their resultant effects on the mechanical performance.

In summary, there have been limited investigations into the effects of residual stress and microstructure on the mechanical property interactions in FSW cross-welded joints. In this study, tensile tests were conducted across different regions of the 2024-T3 cross-welded plate by FSW, and the residual stress distribution was characterized by the X-ray diffraction method. Additionally, electron backscatter diffraction (EBSD) analysis was employed to explore the influence of residual stress and microstructure on the tensile strength of the cross-welded joint, providing a theoretical foundation for the research and practical applications of FSW integral welded panels.

## 2. Welding Procedure

**Figure 2:**
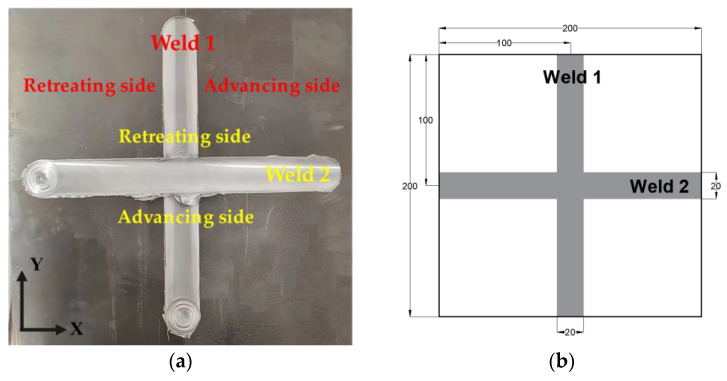
Cross-welded specimen: (**a**) cross-welded plate; (**b**) size of cross-welded specimen.

Rectangular 2024-T3 aluminum plates, measuring 200 mm × 200 mm × 8 mm, were joined by the FSW machine as shown in Figure 2. Weld 1 was initially deposited along the rolling direction, and followed by 90° workpiece rotations for the orthogonal direction of Weld 2. Process parameters maintained constant welding speed (150 mm/min) and tool rotation rate (400 rpm) across both single-weld and cross-weld. The welding tool had a shoulder diameter of 20 mm and a probe diameter of 5 mm, with the probe characterized by a threaded design and measuring 7.5 mm in length. Table 1 displays the chemical compositions of the 2024-T3 alloys.

## 3. Tensile Mechanical Properties

**Figure 3:**
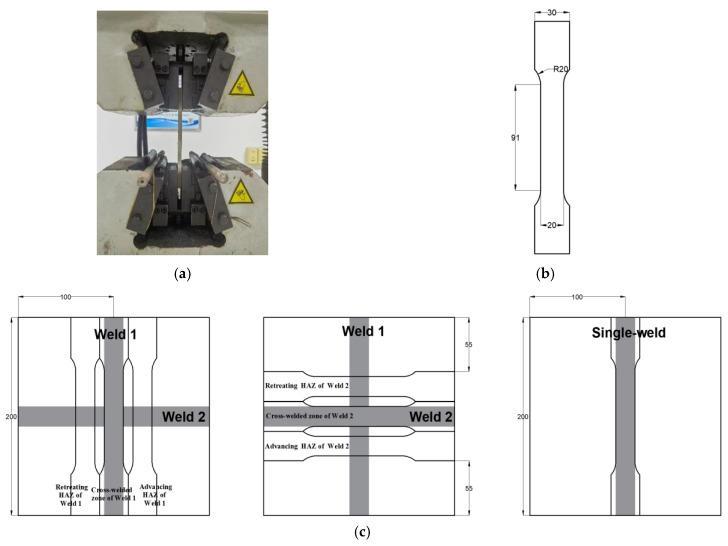
Tensile mechanical experiments: (**a**) tensile test machine; (**b**) size of tensile test samples (mm); (**c**) position of tensile test samples (mm).

The tensile mechanical experiments were conducted using the INSTRON 810 testing machine (Xi’an University of Architecture and Technology, Xi’an, China) with a maximum loading force of 30 kN (Figure 3a). Specimen geometry followed ASTM E8 standards [28] in Figure 3b. As illustrated in Figure 3c, six tensile test specimens were taken from different zones: the advancing HAZ of Weld 1, the cross-welded zone of Weld 1, the retreating HAZ of Weld 1, the retreating HAZ of Weld 2, the cross-welded zone of Weld 2, and the advancing HAZ of Weld 2. In addition, the single-welded specimen was also tested with the same geometry in Figure 3c. To ensure the accuracy of the tensile tests, each sample was tested three times to confirm the validity of the data, and the results were averaged to provide the final tensile values.

**Figure 4:**
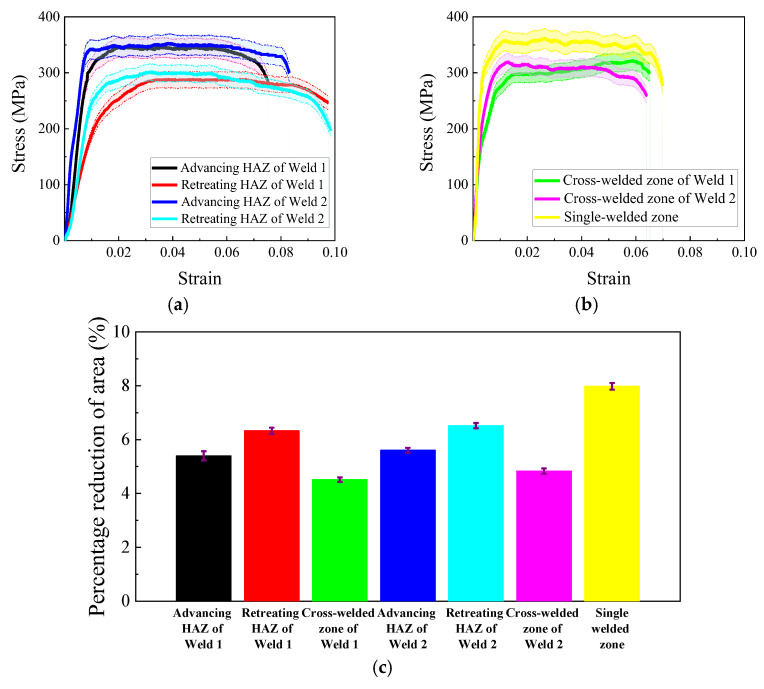
Comparison of tensile mechanical experiments: (**a**) stress–strain curves of HAZ; (**b**) stress–strain curves of WZ; (**c**) percentage reduction in area.

The tensile responses of the cross-welded joints are systematically compared through stress–strain curves in Figure 4a,b. For 2024 aluminum alloy, which lacks a distinct yield plateau, the 0.2% offset method is employed to determine yield strength. The advancing HAZ exhibits superior yield strength compared to the retreating side, with the advancing HAZ of Weld 1 reaching 316.2 MPa versus 226.9 MPa for the retreating side, representing the 28.3% strength differential. Similarly, Weld 2 demonstrates this trend, showing 341.8 MPa and 281.8 MPa for the advancing and retreating HAZ, respectively, corresponding to a 17.6% reduction. The cross-welded zone displays significant strength degradation relative to the single-welded zone. The cross-welded zone of Weld 1 yields at 254.1 MPa, which is 22.2% below the single-welded zone. While the cross-welded zone of Weld 2 shows a 7.9% decrease to 300.4 MPa compared with the single weld.

Percentage reduction in area is a critical parameter in tensile testing that quantifies the ductility of a material by measuring the decrease in cross-sectional area at the point of fracture relative to the original cross-sectional area. Fracture analysis reveals consistent ductility loss in the cross-welded zone, with a percentage reduction in area averaging 4.7% compared to 8.0% for the single-welded zone. Notably, the retreating HAZ exhibits enhanced ductility, particularly in Weld 2, where maximum fracture strain reaches 6.5%. Furthermore, the HAZ of Weld 2 demonstrates greater ductility than that of Weld 1. These mechanical anisotropies correlate with residual stress distributions and microstructure gradients detailed in the subsequent analyses.

**Figure 5:**
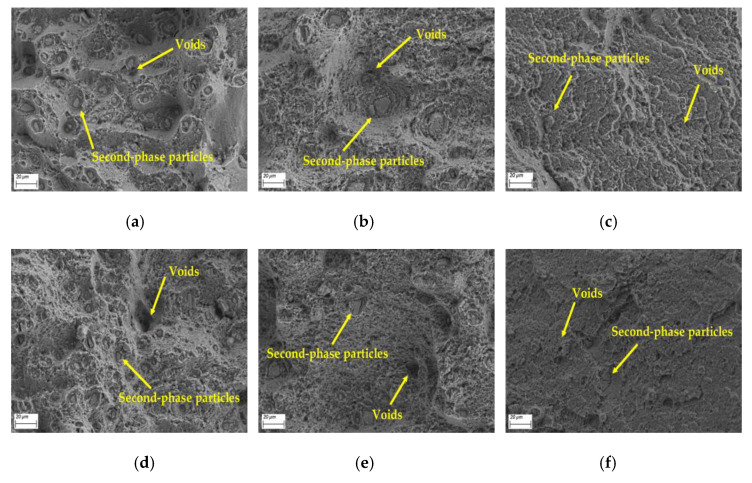
Fracture morphologies of welds: (**a**) the advancing HAZ of Weld 1; (**b**) the retreating HAZ of Weld 1; (**c**) the cross-welded zone; (**d**) the advancing HAZ of Weld 2; (**e**) the retreating HAZ of Weld 2; (**f**) the single-welded zone.

The tensile microstructural characterization via SEM shows critical damage evolution mechanisms in the cross-welded joints. Figure 5 presents fracture surfaces which are normal to the loading axis, exhibiting dimpled rupture features, characteristic of ductile transgranular failure. A comparison of Figure 5a,b,d,e shows that the HAZ displayed heterogeneous damage accumulation. The advancing side appears to have second-phase particles (Al_2_CuMg), which are smaller than the retreating side [29,30]. This particle size dichotomy originates from differential thermal histories. The thermomechanical conditions of the advancing side promote dynamic recrystallization and precipitate dissolution, whereas on the retreating side, thermal profiles permit partial precipitate coarsening. Consequently, the enhanced yield strength in the advancing HAZ in Figure 4a correlates with refined grains. However, the retreating side exhibits more voids and dimples than the advancing side, which explains the higher fracture strain observed on the retreating side. Due to the repeated heat input, the second-phase particle size in Weld 2 is smaller than that in Weld 1 within the same zone. This accounts for the improved ductility of Weld 2 compared to Weld 1, as reflected in the tensile results shown in Figure 4c.

The WZ undergoes dynamic recrystallization driven by intense thermomechanical processing, and promotes complete recrystallization, forming equiaxed grains as documented in prior studies [31]. Comparative microstructural analysis reveals that cross-welded zones exhibit a reduction in Al_2_CuMg precipitate density compared to the single-welded zone, coupled with shallower voids versus the single weld [29]. This morphological evolution aligns with established void growth mechanics in [32], where shallower voids demonstrate lower strain localization capacity due to constrained dislocation pinning effects. Consequently, the cross-welded zone exhibits a reduction in fracture strain relative to the single-welded zone (Figure 4c), directly correlating with their diminished void-mediated plasticity.

## 4. Residual Stress Measurement

**Figure 6:**
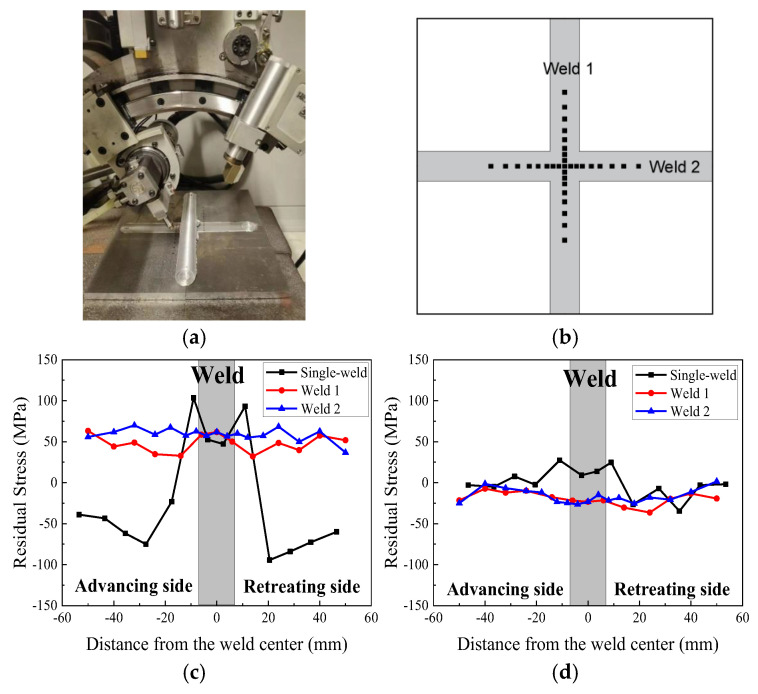
X-ray residual stress measurement: (**a**) X-ray diffraction setup; (**b**) measurement point; (**c**) longitudinal direction measurement results; (**d**) transverse direction measurement results.

The residual stress distribution in the cross-welded and single-welded joints was systematically characterized using the X-350A X-Ray diffraction(Xi’an Jiaotong University, Xi’an, China) (Figure 6a) across predefined measurement points in Figure 6b. Comparative analysis reveals distinct stress state modifications in the cross-welded joint, where the characteristic longitudinal “double peak” stress profile observed in the single-weld is absent due to orthogonal thermal gradient interactions (Figure 6c,d). The cross-welded zone exhibits a 19.12% elevation in longitudinal residual stress compared to the single-welded zone, while the TMAZ in both Weld 1 and Weld 2 demonstrates 32.8–46.9% stress reductions. Longitudinal stress gradients intensify near BM interfaces in Weld 1, contrasting with stress trends in Weld 2. Transverse stress components show near-uniform distributions in Weld 1, with compressive stresses ranging from −23.33 MPa in the weld zone (WZ) to −27.3 MPa in the advancing TMAZ. The cross-welded joint assemblies exhibit a 37 MPa reduction in mean transverse residual stress relative to the single-welded joint. These alterations stem from cross-welded thermomechanical interactions, where secondary thermal cycles from Weld 2 partially annihilate prior dislocation structures in Weld 1, simultaneously constraining plastic deformation in overlapping zones.

## 5. Microstructural Characterization

### 5.1. Microhardness

**Figure 7:**
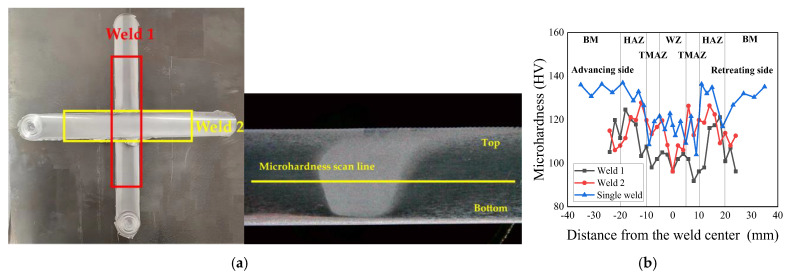
Microhardness distribution of welded joint: (**a**) microhardness measurement position; (**b**) measurement results.

Microhardness mapping is conducted along predefined scan lines in Figure 2 at mid-thickness positions in Figure 7a, revealing significant hardness variations between the single-welded and cross-welded joints. The single-welded joint exhibits superior hardening responses, with the advancing HAZ reaching peak hardness values of 136.9 HV, which is 26.3% and 21.1% higher than corresponding regions in Weld 1 and Weld 2 cross-joints, respectively. The cross-welded zone demonstrates pronounced softening, registering 96.2 HV in the weld center, representing an 18.1% reduction compared to that of the single-welded joint. The lowest microhardness is localized in the retreating TMAZ of Weld 1 at 91.8 HV, correlating with prior observations of relatively more voids in this region. Comparative analysis of sequential welds identified systematic hardening enhancement in Weld 2, particularly within the HAZ and the TMAZ relative to Weld 1. These gradients align with thermal history variations, where the secondary welding cycle in Weld 2 promotes dynamic aging effects that refine precipitate distributions while suppressing dislocation recovery mechanisms. The measured hardness depression in the cross-welded zone inversely correlates with residual stress elevation patterns in Figure 6, suggesting stress-assisted dislocation rearrangement dominates over conventional precipitation strengthening in the cross-welded joint.

### 5.2. EBSD Analysis

**Figure 8:**
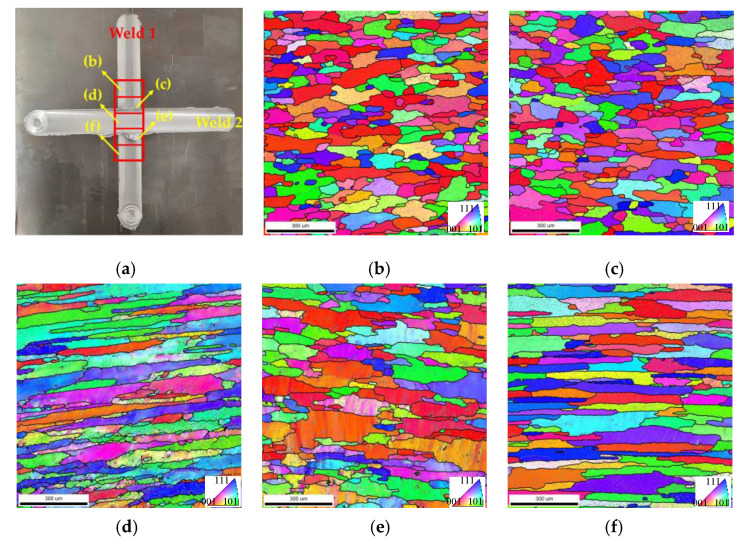
EBSD orientation maps and grain boundaries in Weld 1: (**a**) position of the scan zone in Weld 1; (**b**) retreating side HAZ of Weld 2; (**c**) retreating side TMAZ of Weld 2; (**d**) cross-welded zone; (**e**) advancing side TMAZ of Weld 2; (**f**) advancing side HAZ of Weld 2.

The EBSD analysis in Figure 8 reveals significant microstructural evolution across distinct zones. The EBSD measurement location is depicted in Figure 8a, which position is in the WZ of Weld 1, and represents the retreating HAZ, retreating TMAZ, cross-welded zone, advancing TMAZ, and advancing HAZ of Weld 2, in that order. Orientation maps (Figure 8b–f) demonstrate pronounced grain coarsening in the WZ of Weld 1 compared to the fine equiaxed grains typically observed in the single-welded joints due to secondary thermal cycles during Weld 2 processing [21]. These thermal cycles trigger partial remelting and grain growth, particularly in the advancing regions. Cross-welded zones exhibited elongated grains, which were attributed to shear-dominated deformation during overlapping probe interactions. Mechanical stirring effects suppressed recrystallization kinetics, yielding higher low-angle grain boundary (LAGB, 2–15°) density relative to single-welded counterparts, as evidenced by gray lines in Figure 8d.

**Figure 9:**
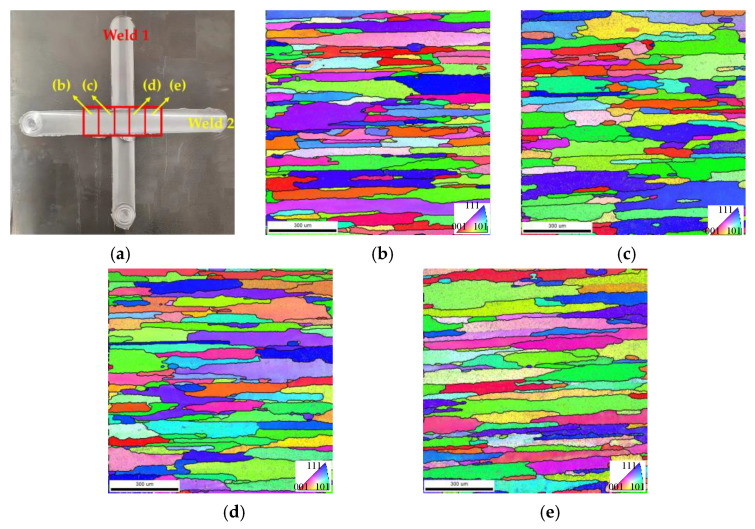
EBSD orientation maps and grain boundaries in Weld 2: (**a**) position of the scan zone in Weld 2; (**b**) retreating HAZ of Weld 1; (**c**) retreating TMAZ of Weld 1; (**d**) advancing TMAZ of Weld 1; (**e**) advancing HAZ of Weld 1.

Figure 9a–e are the EBSD microstructure of Weld 2. The measurement positions are depicted in Figure 9a, which are located in the WZ of Weld 2, and represent the retreating side HAZ, retreating side TMAZ, advancing side TMAZ, and advancing side HAZ of Weld 1, in that order. Orientation mapping demonstrates pronounced grain elongation in the HAZ and TMAZ of Weld 1. This deformation texture, marked by high density LAGBs, indicates incomplete dynamic recrystallization during secondary thermal cycles from Weld 2 processing. Comparative analysis with single-welded joint in [22] showed higher dislocation density in the advancing TMAZ Weld 2, correlating with residual stress concentration patterns observed in Figure 6. These stabilized substructures explain the hardness differentials (Figure 7b) through enhanced dislocation strengthening mechanisms, while simultaneously limiting ductility recovery via constrained grain boundary sliding. The microstructural inheritance effect observed here underscores the critical role of welding sequence in determining the cross-welded joint properties, as prior thermal-mechanical histories dictate subsequent deformation.

**Figure 10:**
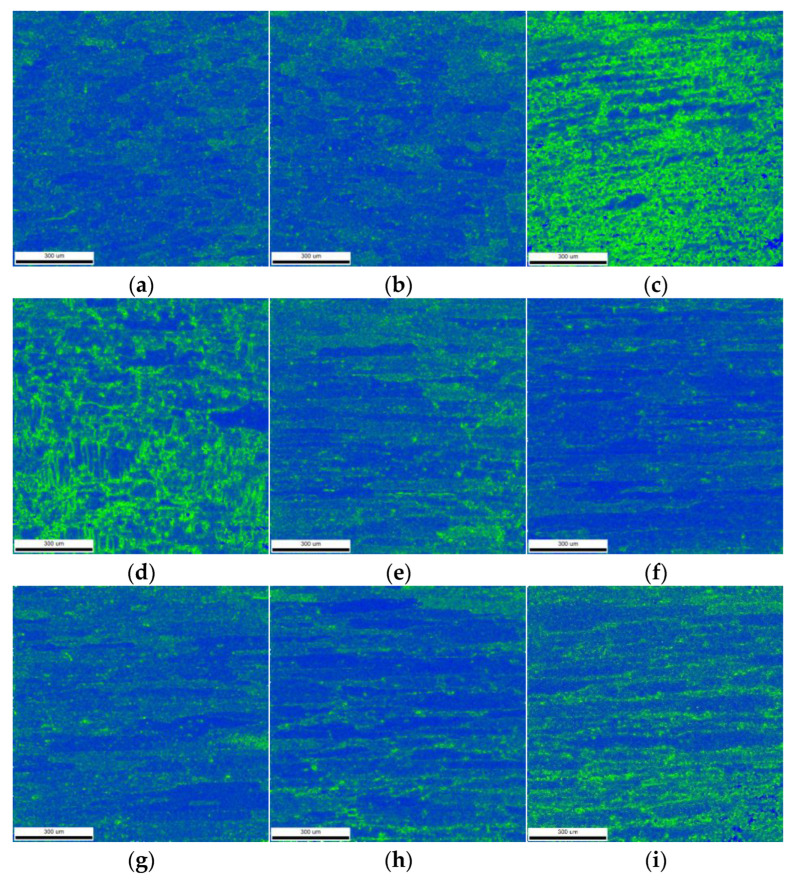
Kernel average misorientation (KAM) maps: (**a**) retreating side HAZ of Weld 2; (**b**) retreating side TMAZ of Weld 2; (**c**) cross-welded zone; (**d**) advancing side TMAZ of Weld 2; (**e**) advancing side HAZ of Weld 2; (**f**) retreating HAZ of Weld 1; (**g**) retreating TMAZ of Weld 1; (**h**) advancing TMAZ of Weld 1; (**i**) advancing HAZ of Weld 1.

Kernel average misorientation (KAM) mapping (Figure 10) quantitatively revealed heterogeneous plastic strain distribution across weld zones, with color visualization delineating deformation gradients. The blue regions denote areas of minimal deformation, whereas the green regions signify areas of maximum deformation. The cross-welded zone and the advancing TMAZ exhibit maximum strain localization, correlating with elevated residual stresses shown in Figure 6c. This strain–stress coupling originates from geometrically necessary dislocation accumulation during overlapping probe interactions, where the advancing side experiences higher shear strain rates than the retreating side due to the tool rotation direction. The intensified plastic flow constraints in the cross-welded zone restrict dislocation annihilation, promoting residual stress amplification through dislocation pileup mechanisms.

The KAM maps in Weld 2 are shown in Figure 10f–i. The WZ exhibits relatively uniform strain distribution due to dynamic recrystallization, generating strain-free grains, while the advancing HAZ demonstrates localized strain concentration exceeding the retreating HAZ. The strain gradient evolution stems from asymmetric thermal cycling during cross welding, where secondary thermal input in Weld 2 partially relaxes prior dislocation structures, while introducing new strain localization through probe induced shear gradients.

**Figure 11:**
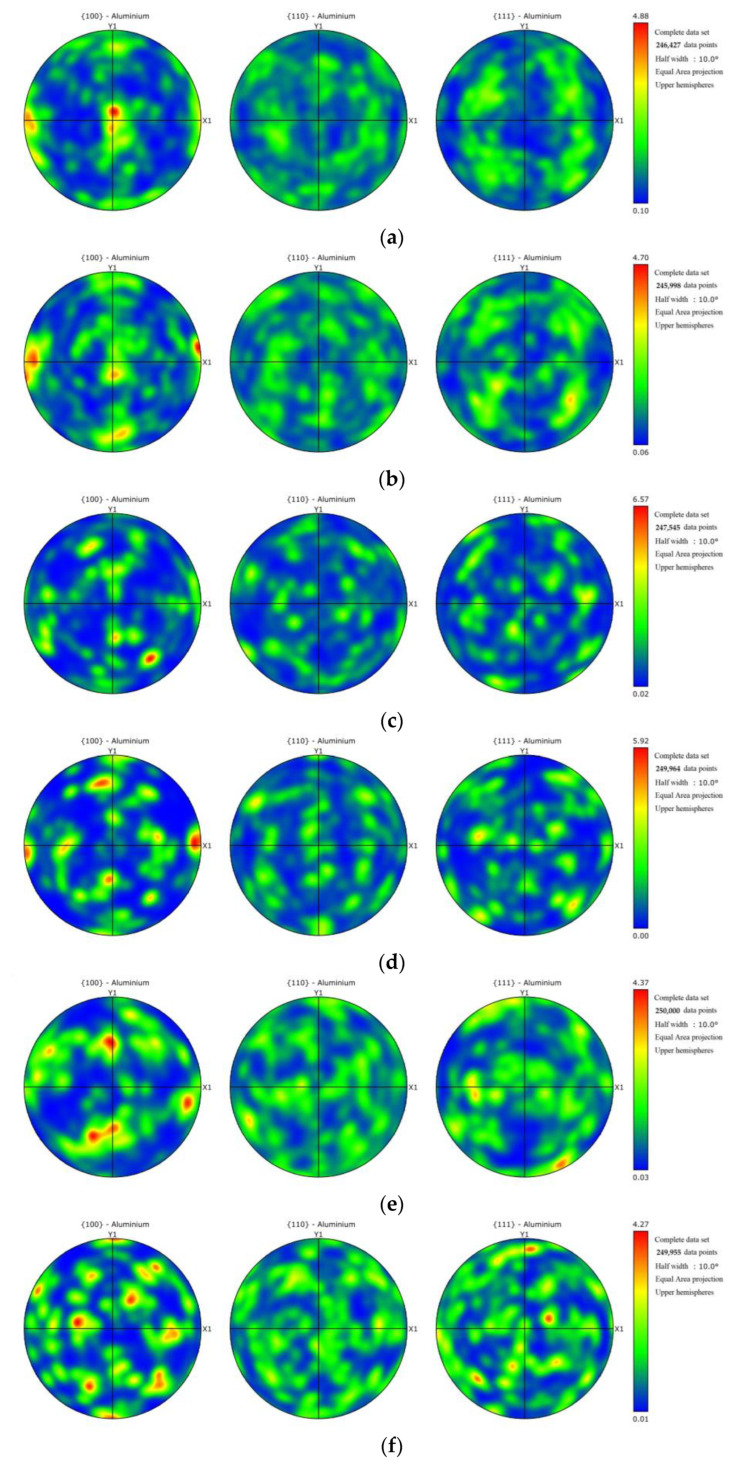

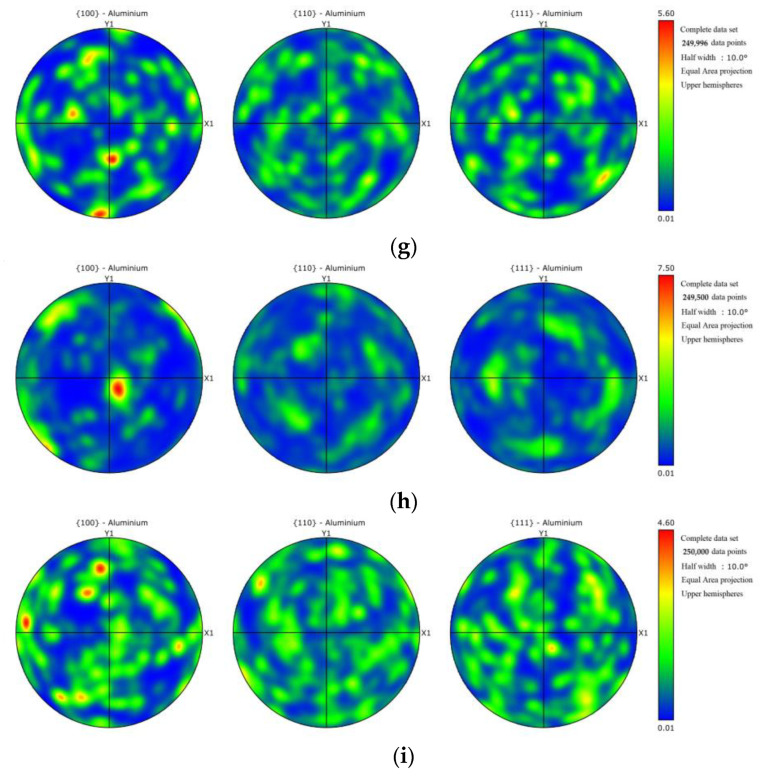
Pole figures: (**a**) retreating side HAZ of Weld 2; (**b**) retreating side TMAZ of Weld 2; (**c**) cross-welded zone; (**d**) advancing side TMAZ of Weld 2; (**e**) advancing side HAZ of Weld 2; (**f**) retreating HAZ of Weld 1; (**g**) retreating TMAZ of Weld 1; (**h**) advancing TMAZ of Weld 1; (**i**) advancing HAZ of Weld 1.

Crystallographic texture evolution in the cross-welded joint was quantified through pole figure analysis (Figure 11), revealing orientation development under the cross-welding process. The {100} and {111} planes demonstrated predominant texture strengths exceeding {110} orientations across all zones, with maximum intensity localized in the advancing TMAZ of Weld 1 (Figure 11h). Conversely, the retreating HAZ of Weld 1 exhibits minimized texture intensity (Figure 11f), reflecting thermal recovery mechanisms that disrupt dislocation patterning. It also shows that dynamic recrystallization in the cross-welded zone continuously generates random equiaxed grains.

## 6. Conclusions

This study systematically elucidates the interdependent relationships between microstructural characteristics, residual stress distributions, and tensile properties in friction stir cross-welded joints, establishing a theoretical foundation for the manufacturing and reliability assessment of 2024 aluminum alloy integral welded panels. The main conclusions are as follows:

(1) The advancing HAZ exhibits superior yield strength compared to the retreating side, and the cross-welded zone displays significant strength degradation relative to the single-welded zone. The ductility in the cross-welded zone is lower than that of the single-welded zone, while the HAZ demonstrates greater ductility compared with the weld zone. The advancing side appears to have second-phase particles, which are smaller than the retreating side. The retreating side exhibits more voids and dimples than the advancing side, which explains the higher fracture strain observed on the retreating side. The cross-welded zones exhibit a reduction in Al_2_CuMg precipitate density compared to the single-welded zone, coupled with shallower voids versus the single weld.
(2) The longitudinal direction residual stress profile of the cross-welded joint does not exhibit the typical “double peak” characteristic. The cross-welded zone shows an elevation in longitudinal residual stress compared to the single-welded zone, while the TMAZ in both Weld 1 and Weld 2 demonstrates stress reductions. Transverse stress components present near-uniform distributions in Weld 1.
(3) The cross-welded joint exhibits lower hardening responses, and the lowest microhardness is localized in the retreating TMAZ of Weld 1. Cross-welded zones exhibited elongated grains, which were attributed to shear-dominated deformation during overlapping probe interactions. The advancing TMAZ and the cross-welded zone exhibit maximum strain localization, correlating with elevated residual stresses. In Weld 2, The WZ exhibits relatively uniform strain distribution due to dynamic recrystallization, generating strain-free grains, while the advancing HAZ demonstrates localized strain concentration exceeding the retreating HAZ.

## Figures and Tables

**Table 1:** Chemical compositions of the 2024-T3 alloys.

Material	Chemical Composition (%)								
2024-T3	Cu	Si	Fe	Mn	Mg	Zn	Cr	Ti	Al
	3.8–4.9	0.5	0.5	0.3–0.9	1.2–1.8	0.25	0.1	0.15	Base

## Data Availability

The original contributions presented in the study are included in the article, further inquiries can be directed to the corresponding author.
